# RETRACTED: Abdel-Shafi et al. Characterization and Antibacterial Activity of 7S and 11S Globulins Isolated from Cowpea Seed Protein. *Molecules* 2019, *24*, 1082

**DOI:** 10.3390/molecules31111853

**Published:** 2026-05-28

**Authors:** Seham Abdel-Shafi, Abdul-Raouf Al-Mohammadi, Ali Osman, Gamal Enan, Samar Abdel-Hameid, Mahmoud Sitohy

**Affiliations:** 1 Botany Department, Faculty of Science, Zagazig University, Zagazig 44519, Egypt; 2 Department of Science, King Khalid Military Academy, P.O. Box 22140, Riyadh 11495, Saudi Arabia; 3 Biochemistry Department, Faculty of Agriculture, Zagazig University, Zagazig 44511, Egypt

The journal retracts the article titled “Characterization and Antibacterial Activity of 7S and 11S Globulins Isolated from Cowpea Seed Protein” [1], cited above.

Following publication, concerns were brought to the attention of the Editorial Office regarding the integrity of an image presented in this publication [1].

In accordance with the journal’s standard procedures, the Editorial Office and Editorial Board conducted an investigation that identified indications of inappropriate editing in Figure 1A. Although the authors cooperated with the investigation, the explanations and supporting materials provided were not deemed sufficient by the Editorial Board to resolve the identified concerns. Consequently, the Editorial Board has lost confidence in the reliability of the findings and decided to retract this publication, as per MDPI’s retraction policy.

This retraction was approved by the Editor-in-Chief of the *Molecules* journal.

The authors have been informed of this retraction and have indicated their disagreement with this decision.
